# Social Networks and Their Influence on the Choice of Unassisted Smoking Cessation: Cross-Sectional Study in Six Cities in China

**DOI:** 10.2196/74147

**Published:** 2026-03-25

**Authors:** Sihui Peng, Tingzhong Yang, Lijing Li, Weifang Zhang, Randall R Cottrell

**Affiliations:** 1 School of Medicine Jinan University Guangzhou China; 2 Center for Tobacco Control Research Zhejiang University Hangzhou, Zhejiang China; 3 Research Center for Digital Health Behavior Theory and Management National Health Big Data Institute Zhejiang University Hangzhou China; 4 Injury Control Research Center West Virginia University Morgantown, WV United States; 5 Department of Obstetrics and Gynecology Yongkang Women and Children’s Health Hospital Yongkang, Zhejiang China; 6 Children's Hospital of Zhejiang University Hangzhou, Zhejiang China; 7 Public Health Studies Program School of Health and Applied Human Sciences University of North Carolina Wilmington, NC United States

**Keywords:** unassisted smoking cessation, smoking cessation, social network, tobacco control, smoking, smoke, ordinary social network, lower social network, higher social network

## Abstract

**Background:**

Many countries, including China, have implemented nationwide smoking cessation programs in accordance with Article 14 of the World Health Organization (WHO) Framework Convention on Tobacco Control (FCTC). However, the use of assisted smoking cessation services remains low, while unassisted smoking cessation (USC) methods are widely preferred. Studies have shown that many individual and environmental factors are associated with USC adoption. However, no studies have examined the association between social networks and USC adoption.

**Objective:**

This study examined the effects of social networks on USC choice among male smokers in China.

**Methods:**

A cross-sectional multivariable sampling design was used to interview subjects from six selected cities in China. The study sample included only male participants. A standardized questionnaire was used to obtain information about sociodemographic characteristics, social networks, and USC choice. Multivariable logistic regression models were used to examine the association between social networks and USC choice. Furthermore, quantitative analysis was conducted to demonstrate the dose-response relationship between the city-level social networks and the prevalence of USC. Structural equation modeling (SEM) was used to establish the mechanisms by which reference group norms affect USC through social networks.

**Results:**

We identified 2852 smokers, of whom 1647 (57.7%) had attempted to or had quit smoking. Among them, 91.6% (n=1509; 95% CI 90.9%-97.5%) reported quitting without assistance, and 42.1% (n=58; 95% CI 32.4%-61.3%) of the remaining 138 (8.4%) participants who used USC methods achieved abstinence. Multiple logistic regression analysis found that although the higher social network (HSN) is not significantly associated with USC adoption, both the ordinary social network (OSN) and the lower social network (LSN) are significantly negatively associated with USC adoption. Compared to the low-size group of the OSN, the odds ratios (ORs) for the medium-, high-, and very high-size groups were 0.23 (95% CI 0.10-0.80), 0.22 (95% CI 0.07-0.60), and 0.25 (95% CI 0.08-0.72), respectively. Similarly, compared to the low- and medium-size groups of the LSN, the OR for the high-size group was 0.32 (95% CI 0.23-0.45). The analysis revealed a significant positive dose-response relationship of city-level OSN and LSN sizes with the probability of USC adoption, as indicated by regression coefficients β=0.2784 (P<.01) and β=2.2269 (P<.01), respectively. SEM analysis indicated that the size of OSNs and LSNs exerts an indirect effect on the relationship between reference group norms and USC (β=0.2315, P<.01; β=0.4613, P<.01).

**Conclusions:**

Both OSNs and LSNs are significantly negatively associated with USC adoption. The findings underscore the significance of USC and its potential role in reducing smoking prevalence within the Chinese population.

## Introduction

Numerous studies have shown that the success rate of smoking cessation assisted by health professionals, which includes both pharmacological and nonpharmacological methods, is higher than that of unassisted smoking cessation (USC) [1-3]. Article 14 of the World Health Organization (WHO) Framework Convention on Tobacco Control (FCTC) requires parties to promote smoking cessation and enforce effective measures to assist tobacco users in quitting [4]. For decades, various countries worldwide have made efforts to assist people with smoking cessation. In China, nationwide smoking cessation clinics and hotlines have been established by the central and numerous local governments, but the number of smokers who use these smoking cessation services is small [5]. In contrast, USC methods are widely used by individuals seeking to quit smoking, as they believe they can achieve abstinence through their own efforts without the need for external assistance [6-8].

To understand why this situation occurs, a thorough understanding of the nature of USC is necessary. Studies have shown that some individual and environmental factors are associated with USC adoption [6,9-11]. When considering environmental influences on USC adoption, the environment in which people live is primarily shaped by the interactions between individuals, their social relationships, and social networks. Interpersonal relationships and social networks, as key environmental variables, are likely to have a significant impact on people’s adoption of USC.

The theory of reasoned action (TRA) offers a valuable framework for understanding individual decision-making in health-related behaviors, including smoking cessation. According to TRA, behavioral intention is shaped by two primary factors, attitudes toward the behavior and subjective norms [12]. In the context of USC, these referent norms may encompass beliefs and social pressures from close contacts, such as family members, peers, or colleagues. TRA further suggests that normative beliefs, behavioral beliefs, and attitudes, especially those shaped by individuals with whom the person shares meaningful relationships, can significantly impact behavioral decisions [12]. Accordingly, the structure and composition of one’s social network may play a pivotal role in shaping the intention to quit smoking without external aids. For example, individuals embedded in networks where smoking is discouraged or where others have successfully quit may be more inclined to attempt smoking cessation independently. In contrast, networks that normalize smoking or lack smoking cessation role models may diminish the likelihood of unassisted quitting. Building upon this theoretical foundation, our study examined how social network size and characteristics relate to the choice of USC. We hypothesized that broader or more supportive networks may facilitate autonomous quitting decisions, in line with the mechanisms proposed by TRA.

The concept of social networks emphasizes that each individual is connected to others, who, in turn, are connected to many more. Individuals linked through social ties influence one another’s norms, attitudes, and behaviors. When people are embedded in networks with smokers, repeated exposure to smoking behavior can normalize it to the extent that smoking becomes a perceived social norm. This norm is further reinforced by others who smoke within the network. Social networks serve as contexts for communication, reinforcement of beliefs, and daily interactions. Scholars have argued that strong ties within a network possess a unique capacity to shape beliefs and behaviors, while weak ties facilitate the diffusion of diverse information and behavioral alternatives. Strong ties tend to promote conformity to existing norms [13,14]. Empirical studies have shown that adolescents who occupy central positions within social cliques tend to exhibit lower-than-average smoking rates, whereas those who are socially isolated often display higher-than-average smoking rates [15-18]. People’s daily life involves dealing with various relationships, such as those with themselves, with nature, with the supernatural, and with others. Chinese culture places great emphasis on interpersonal relationships, considering them perhaps the most important aspect of their life [12]. With the rapid economic development in China over the past 40 years, managing social interactions and personal relationships has become increasingly important. It serves as a foundation for the commercial enterprise and business acumen that are deeply ingrained in society. Chinese culture regards smoking as a tool for social interaction [19]. Studies have reported that in China, the most important reasons for smoking are embedded in social interactions and interpersonal relationships [20,21]. In Chinese culture, offering a cigarette has been a custom to strengthen personal friendships and relationships. Chinese people share cigarettes with one another and offer them to guests as a sign of hospitality [19].USC is a self-determined, self-help smoking cessation behavior that is inherently closely linked to social networks. The influence of social networks on USC may be particularly prominent in Chinese culture.

Social network theory emphasizes the fact that the structural and compositional features of a social network are its essential functions. Social networks matter because different types of connections have different effects on behaviors. The impact on smoking by networks composed of different types of ties—family versus friends, distant versus proximate—is complicated and can be different for different individuals. Some studies have found smoking cessation among social networks members, including spouses, siblings, friends, and neighbors, are particularly relevant for smoking cessation [22-25]. Therefore, based on these findings, we hypothesized that the type of social network is positively associated with the adoption of USC.

No studies have examined the association between social networks and USC adoption. This study aimed to explore the impact of social networks on USC adoption. The findings should help in understanding the nature of USC and its role in reducing smoking prevalence among the Chinese population. As most people quit smoking through the use of USC, we believe that USC can serve as a proxy for the common characteristics of smoking cessation.

Evidence from several studies suggests that USC may function as a scalable public health strategy, capable of lowering smoking rates across the broader population [6]. A study [26] found that unassisted quitting contributed to successful quitting by approximately 70 million smokers in China. This effectively decreased the smoking prevalence in the population and yielded substantial health benefits. These findings contribute to a deeper understanding of a key factor affecting USC trends at the population level, particularly in settings where assisted methods are less accessible or culturally less adopted.

According to TRA, reference group norms and social expectations significantly affect behavioral intentions. In this study, we used structural equation modeling (SEM) to establish the mechanisms by which reference group norms affect USC through social networks. Reference group norms not only exert a direct effect on USC but also indirectly shape this behavior through the size of general and lower-level social networks. The more individuals one interacts with in these networks, the stronger the normative the pressures become, thereby amplifying the impact of subjective norms on smoking cessation outcomes. SEM is particularly appropriate in this context, as it allows for the simultaneous estimation of direct and indirect pathways, providing a more nuanced understanding of the interplay between individual perceptions and social structures. This approach enriches the explanatory power of TRA by integrating social network dynamics, and it offers practical insights for designing interventions: by identifying key network structures and normative influences, policymakers and health practitioners can more effectively promote smoking cessation behaviors.

This study sought to address the following research questions:

- How do variations in the social network size influence USC?
- What is the quantitative relationship between regional-level differences in the social network size and USC?
- Do different social network sizes serve as mediators between subjective norms and USC?

## Methods

### Study Design

This observational, cross-sectional study had a multistage cluster sampling design. First, we selected six sample cities from various regions in China and then two residential districts from the primary urban areas within each city. Next, we selected four communities from each district and then five building blocks within each community. Finally, within each building block, we selected 1 out of every 20 households on the family household registration list. All selection was random.

### Data Collection

Participants were identified based on specific criteria set by the research team. Inclusion criteria were as follows: (1) the male resident in the household (if there were two or more male residents, the person whose birth date was closest to the contact date was selected); (2) age≥15 years; (3) living in the study city for at least 1 year; and (4) willingness to participate in the study and provide survey-based information. Individuals were excluded if they refused to provide survey-based information or had a medical condition that could limit or preclude their participation.

Potential participants were approached by trained interviewers to gauge their willingness to participate. Each interviewer received 1-day training on the survey protocol and interviewing procedures.

A self-administered questionnaire was scheduled once an individual agreed to participate in the survey. The questionnaire was administered by fourth-year medical students or graduates from a local medical college. The survey protocol was identical across all six cities to maintain consistency in interviews and data collection. Participants were privately administered the survey in their homes or at a quiet designated location, such as a backyard or a community park. The survey primarily took place on weekends, in the evening, or at other times convenient for the participants.

### Ethical Considerations

This study adhered to the guidelines stated in the World Medical Association’s Declaration of Helsinki. Ethical approval was obtained from the Ethics Committee at the Medical Center of Zhejiang University (approval number 2014:1-017). Verbal informed consent was obtained from all participants before data collection. Participants were provided with clear and detailed information about the study and informed that their participation was voluntary and that they could withdraw at any time without consequence. All responses were kept anonymous, and each participant got an opportunity to ask for clarification about the survey questions. The survey data were securely stored in accordance with institutional guidelines, with access restricted to authorized personnel only. Participants typically spent around 10 minutes completing the survey. After a qualification check, they received a gift of renminbi (RMB) 10 (~US $1.46) after questionnaire completion.

### Measurements

#### Dependent Variables

Participants were asked whether they currently smoked. Response options included the following: “Yes, smoke every day; “Yes, smoke on one or more days but not every day”; and “No.” Those who answered yes were categorized as current smokers. Those who quit smoking were defined as individuals with a continuous or cumulative smoking history of at least 6 months or more who had abstained from smoking at the time of the study. Smoking intensity consisted of smoking amount and smoking duration. Quitting attempts were defined as having made at least three attempts to quit smoking, each lasting a minimum of 3 days, without achieving sustained abstinence at the time of the survey [10].

USC is defined as smokers quitting on their own without professionally or pharmacologically mediated assistance; however, USC includes the use of over-the-counter agents, such as nicotine gum and patches. USC attempts refer to smoking cessation efforts made without professional support. USC success denotes cases in which individuals achieve sustained abstinence without using interventions such as guidance from health professionals, quitting clinics, hotlines, or other structured support resources [11,27].

#### Independent Variables

In this study, we measured the social network size using the network scale-up method. Participants were asked via surveys how many people they know from different subpopulations [28,29]. We created our questionnaire by appropriately considering Chinese culture and societal norms and making modifications accordingly. The questionnaire included 16 items under “total social network” (TSN) categorized into three subquestionnaires: (1) ordinary social network (OSN), which consisted of 6 items and asked “How many people do you know with the surname Ma, Zhu, or another surname?”; (2) lower social network (LSN), which consisted of 3 items and asked “How many people do you know who are seriously ill, victims of domestic violence, or drug users?”; and (3) higher social network (HSN), which consisted of 5 items and asked “How many people do you know who are company managers, professors, engineers, party and government officials, or physicians?” The network sizes were estimated by directly adding the number individuals known to participants [12].

We conducted comprehensive reliability and validity analyses for the TSN and its three subcomponents: OSN, HSN, and LSN. The TSN demonstrated excellent internal consistency, with Cronbach α=.91. Exploratory factor analysis (EFA) was performed on 14 observed variables, resulting in the extraction of three distinct factors. The item groupings within each factor corresponded closely with the theoretical expectations. Specifically, the OSN factor explained 33.5% of the total variance; the HSN factor, 12.2%; and the LSN factor, 9.8%, yielding a cumulative explained variance of 55.5%. This level of explained variance is considered acceptable, particularly in exploratory studies, where a threshold of 50% or higher is commonly recommended [30,31]. All items exhibited loadings above 0.65 within the OSN factor, above 0.59 within the HSN factor, and above 0.42 within the LSN factor. These values met or surpassed the commonly accepted threshold of 0.40 for factor loadings, which is widely used to assess construct validity in EFA [31,32].

In addition, EFA was conducted separately for each subscale. Each subscale yielded a single underlying factor, which explained 59% of the variance for the OSN, 50% for the HSN, and 56% for the LSN. According to established methodological guidelines, a single factor that accounts for at least 40% of the total variance is generally considered acceptable in exploratory research within the social sciences [31]. All items exhibited factor loadings above 0.48 in the OSN measure, above 0.47 in the HSN measure, and above 0.44 in the LSN measure. Factor loadings above 0.40 are generally considered acceptable indicators of construct validity [31,32]. In this study, all retained items met or surpassed this threshold, indicating satisfactory construct representation across all three subscales. Cronbach α for the OSN, HSN, and LSN subscales was .82, .68, and .79, respectively. Although Cronbach α for the LSN fell slightly below the conventional threshold of .70, it was sufficiently close to be deemed acceptable, particularly in our exploratory research settings, where slightly lower reliability is often tolerated [31-33]. All social network measures demonstrated acceptable reliability and construct validity, supporting the robustness of the measurement framework.

Within the TRA framework, several variables were included: social norms for smoking cessation, OSNs, LSNs, and USC. The latter three have already been described in this paper; social norms for smoking cessation were measured with the question, “Have any of your five closest friends quit smoking?” with response options coded as “Yes” and “No.”

#### Covariates

Sociodemographic characteristics were age, ethnicity, educational level, occupation, family location, and household income. Family location was where the participants’ families were located and was categorized into three types: rural area or township, county town or county-level city, and medium or large city. Household income data were collected by requesting participants to report the average income per person in their household for the previous year.

### Statistical Analysis

All data were entered into a database using Microsoft Excel. The dataset was then imported into SAS (9.4 version) for statistical analyses. Descriptive statistics were calculated for USC adoption and success prevalence. Unadjusted logistic models were built for each primary predictor. SAS survey logistic procedures were applied in the analysis. The district was used as the clustering unit in order to account for a within-clustering correlation, attributable to the complex sample for analysis. Multiple logistic regression analysis was conducted to examine the association between social networks and USC adoption.

We constructed several models for the multivariable logistic regression analyses. For USC adoption, the models were (1) the base model, which included individual demographic variables to form (2) the demographic model, in which we entered the number of cigarettes smoked and different types of social networks to form (3) the number-of-cigarettes-smoked model, (4) the OSN model, (5) the LSN model, and (6) a comprehensive model encompassing all these.

To evaluate multicollinearity among the independent variables, variance inflation factors (VIFs) were calculated using standard linear regression procedures. Each predictor was included in the comprehensive model, and VIF values were computed to quantify the extent to which the variance of each estimated coefficient was inflated due to correlations with other predictors. A VIF greater than 10 is generally considered indicative of severe multicollinearity, whereas values below 5 are typically regarded as acceptable, suggesting no serious multicollinearity concerns [34].

Model fit was evaluated using multiple information criteria, including −2 log likelihood, the Akaike Information Criterion (AIC), and the Schwarz Criterion (SC). Lower values of these indices indicate better model fit and were used to compare both nested and nonnested models. To assess potential overdispersion in the multivariable logistic regression models, the Pearson chi-square statistic divided by its degrees of freedom (*χ*²/*df*) was examined. A *χ*²/*df* ratio substantially greater than 1 is commonly interpreted as evidence of overdispersion, suggesting that the observed variability exceeds expectations under the assumed binomial distribution [35]. Conversely, a *χ*²/*df* ratio below 3 is generally considered indicative of acceptable model fit and reasonable residual variance [36].

Subsequently, we applied SEM using the CALIS procedure in SAS to investigate the effects of reference group norms and social networks on USC. The analysis specifically examined whether reference group norms have a direct impact on USC or whether their influence is mediated through OSNs and LSNs. SEM, as a second-generation multivariate technique, allows for the simultaneous estimation of causal relationships among multiple variables. Parameter estimates were obtained using the maximum likelihood method, and model fit was assessed with standard indices, including the goodness-of-fit index (GFI>0.90), the normed fit index (NFI>0.90), and the root mean square error of approximation (RMSEA<0.10) [37].

All analyses were weighted. The weights included (1) sampling weights, calculated as the inverse of the probability of selection at the city and district levels and then multiplied together; (2) nonresponse weights, which accounted for household and individual factors; and (3) poststratification weights, derived using age groups (<25, 25-34, 35-44, 45-54, and ≥55 years) based on the estimated distributions of these characteristics from a national survey [32]. The final overall weights were computed as the product of these three weight sets.

## Results

### Participant Details

A total of 6500 individuals were identified as potential participants for this study, of whom 6010 (93.9%) agreed to participate, when contacted. Of the 6010 questionnaires, 5782 (96.2%) valid records were obtained. Participants’ demographic characteristics are presented in Table 1. Of the 5782 participants, 2852 (49.3%) were smokers (95% CI 41.1%-48.5%). Among current smokers, 1647 (57.7%) had attempted to or had quit smoking, of whom 91.6% (n=1509; 95% CI 90.9%-97.5%) reported quitting without assistance, and 42.1% (n=58; 95% CI 32.4%-61.3%) of the remaining 138 (8.4%) participants who used USC methods achieved abstinence. Unadjusted logistic regression analysis showed that age, education level, marital status, occupation, number of cigarettes smoked, smoking duration, OSNs, and LSNs are significantly associated with USC adoption (see Table 1).

**Table 1:** Characteristics of participants who attempted to or had quit smoking (N=1647) and USC^a^ prevalence.

Characteristics		Participants, n (%)	USC prevalence (%)	Unadjusted OR^b^ (95% CI)
**Age (years)**				
	<25	155 (9.8)	83.6	1.00
	25-34	315 (16.8)	88.1	1.45 (0.98-2.99)
	35-44	427 (19.4)	90.0	1.78 (0.88-3.61)
	45-54	406 (23.3)	92.8	2.51 (1.94-3.25)^c^
	≥55	344 (30.7)	96.1	4.78 (2.16-10.15)^c^
**Ethnicity**				
	Han	1566 (95.6)	92.0	1.00
	Minority	81 (4.4)	81.5	0.38 (0.16-0.92)^d^
**Education**				
	Elementary school or less	158 (17.0)	98.8	1.00
	Junior high school	434 (29.9)	90.3	0.12 (0.03-0.50)^c^
	High school	481 (17.4)	91.2	0.09 (0.02-0.42)^c^
	Junior college	344 (17.4)	91.4	0.13 (0.05-0.37)^c^
	College or more	230 (12.2)	91.2	0.13 (0.03-0.68)^d^
**Marital status**				
	Unmarried	298 (18.1)	86.3	1.00
	Married	1248 (75.6)	92.4	1.94 (1.31-2.88)^c^
	Divorced or widowed	101 (6.4)	96.1	3.86 (0.99-14.89)
**Occupation**				
	Manager and service	208 (8.7)	95.0	1.00
	Professional	140 (8.3)	87.6	0.37 (0.19-0.78)^c^
	Commercial and social service	321 (18.4)	91.3	0.55 (0.21-1.47)
	Technical worker	492 (29.7)	91.5	0.57 (0.25-1.29)
	Operations	188 (15.7)	93.5	1.47 (0.46-4.71)
	Retired	59 (3.9)	92.5	0.54 (0.27-1.07)
	Student	74 (4.9)	90.7	0.49(0.15-1.61)
	Other	165 (10.2)	94.7	0.32(0.10-1.01)
**Income/person/year (RMB^e^; US $^f^)**				
	<20,000; 2923.38	489 (30.1)	92.3	1.00
	20,000-39,999; 2923.38-5846.61	504 (31.6)	91.3	0.88 (0.39-2.02)
	40,000-59,999; 5846.76-8769.99	288 (16.2)	91.0	0.84 (0.41-1.72)
	≥60,000; 8770.13	366 (22.0)	91.4	0.89 (0.57-1.41)
**Number of cigarettes smoked**				
	<10	875 (56.5)	95.0	1.00
	10-19	372 (18.3)	92.7	0.67 (0.31-1.42)
	≥20	400 (25.2)	83.1	0.26 (0.14-0.49)^c^
**Smoking time(year)**				
	<10	764 (51.7	93.6	1.00
	10-19	194 (14.7	86.4	0.43 (0.18-1.10)
	20-29	300 (14.8	90.3	0.64 (0.28-1.47)
	≥30	289 (18.7	91.2	0.72 (0.41-1.25)
**TSN^g^ size**				
	<10	204 (14.8)	94.2	1.00
	10-19	466 (26.6)	93.3	0.86 (0.36-2.05)
	20-29	428 (25.3)	90.0	0.62 (0.34-1.11)
	≥30	549 (33.3)	89.4	0.53 (0.27-1.04)
**OSN^h^ size**				
	<10	218 (15.1)	97.3	1.00
	10-14	448 (25.5)	91.2	0.29 (0.08-0.96)^d^
	15-19	483 (30.0)	89.5	0.23 (0.08-0.72)^c^
	≥20	498 (29.4)	91.0	0.28 (0.09-0.84)^d^
**LSN^i^ size**				
	0	773 (48.6)	94.5	1.00
	1-2	512 (29.0)	93.0	0.76 (0.43-1.40)
	≥3	362 (22.4)	83.4	0.29 (0.18-0.46)^c^
**HSN^j^ size**				
	<5	339 (29.0)	92.4	1.00
	5-9	309 (26.0)	92.9	1.07 (0.57-2.01)
	10-14	224 (26.0)	89.8	0.71 (0.59-1.33)
	≥15	240 (24.0)	90.2	0.87 (0.31-1.47)

^a^USC: unassisted smoking cessation.

^b^OR: odds ratio.

^c^*P*<.01.

^d^*P*<.05.

^e^RMB: renminbi.

^f^The currency exchange rate applied was RMB 1=US $0.15.

^g^TSN: total social network.

^h^OSN: ordinary social network.

^i^LSN: lower social network.

^j^HSN: higher social network.

VIF analysis revealed that all variables had values below 5, ranging from a minimum of 1.3 to a maximum of 4.7, which are generally considered acceptable. These results support the reliability of the regression estimates and indicate that multicollinearity is not a major concern in the current model specification.

Model fit statistics indicated progressive improvement from the demographic model to the OSN model and the LSN model. The −2 log likelihood decreased from 3534.6 in the demographic model to 3456.7 in the OSN model and 3461.5 in the LSN model, accompanied by corresponding reductions in the AIC and SC values, with the former outperforming the latter. These results suggest that the inclusion of social network covariates substantially enhanced model fit. Notably, the *χ*²/*df* ratio decreased across models: 1.32 for the base model, 0.96 for the demographic model, and 0.93 for the comprehensive model. These values indicated satisfactory model fit and suggested that the overall residual variation was within an acceptable range.

Multiple logistic regression analysis revealed that older age was linked to increased adoption of USC in the 45-54–year age group (odds ratio [OR] 2.16, 95% CI 1.05-4.45) compared to the <25-year age group (see Table 2). Those in junior high school exhibited lower USC adoption (OR 0.57, 95% CI 0.39-0.85) compared to the reference group. The number of cigarettes smoked was significantly correlated with lower adoption of USC in the 10-19-cigarette (OR 0.66, 95% CI 0.44-0.97) and 20-cigarette (OR 0.51, 95% CI 0.41-0.63) groups. Higher OSN sizes were significantly associated with lower adoption of USC (OR 0.23, 95% CI 0.10-0.80), (OR 0.22, 95% CI 0.07-0.60), and (OR 0.25, 95% CI 0.08-0.72). Similarly, a high LSN size was significantly associated with lower adoption of USC 0.32 (95% CI 0.23-0.45).

**Table 2:** Multivariable analysis of social net position and USC^a^ adoption.

Characteristics		Demographic model	Number-of-cigarettes-smoked model, OR^b^ (95% CI)	OSN^c^ model, OR (95% CI)	LSN^d^ model, OR (95% CI)	Comprehensive model, OR (95% CI)
**Age (years)**						
	<25	1.00	1.00	1.00	1.00	1.00
	25-34	1.43 (0.54-3.77)	1.91 (1.14-3.20)^e^	2.08 (1.28-3.39)^e^	1.88 (1.08-3.29)^f^	1.64 (1.03-2.52)^f^
	35-44	0.89 (0.36-2.21)	1.23 (0.86-1.74)	2.43 (1.04-5.56)^f^	2.25 (0.92-5.20)	2.06 (1.20-3.53)^f^
	45-54	2.16 (1.05-4.45)^f^	2.99 (1.68-5.33)^e^	3.78 (2.45-5.83)^e^	3.00 (1.84-4.74)^e^	3.15 (2.03-4.91)^e^
	≥55	1.66 (0.81-3.43)	2.06 (1.51-2.83)^e^	4.52 (1.77-11.52)^e^	4.01 (1.47-10.89)^e^	4.43 (1.57-11.31)^e^
**Education**						
	Elementary school or less	1.00	1.00	1.00	1.00	1.00
	Junior high school	0.57 (0.39-0.85)^e^	0.60 (0.38-0.95)^f^	0.14 (0.03-0.64)^e^	0.14 (0.03-0.68)^f^	0.13 (0.02-0.76)^f^
	High school	1.37 (0.60-3.15)	1.50 (0.55-4.09)	0.15 (0.03-0.75)^f^	0.15 (0.03-0.74)^f^	0.12 (0.02-0.78)^f^
	Junior college	1.04 (0.30-3.65)	1.08 (0.27-4.30)	0.19 (0.05-0.69)^f^	0.20 (0.06-0.69)^f^	0.18 (0.04-0.71)^f^
	College and more	0.96 (0.77-1.18)	0.90 (0.69-1.17)	0.21 (0.03-1.57)	0.22 (0.03-1.54)	0.19 (0.02-1.75)
**Number of cigarettes smoked**						
	<10	—^g^	1.00	1.00	1.00	1.00
	10-19	—	0.66 (0.44-0.97)^f^	0.81 (0.45-1.47）	0.74 (0.41-1.35）	0.67 (0.37-1.22)
	≥20	—	0.51 (0.41-0.63)^e^	0.25 (0.12-0.52)^e^	0.26 (0.12-0.57)^e^	0.24 (0.11-0.59)^e^
**OSN size**						
	<10 (low)	—	—	1.00	—	1.00
	10-14 (medium)	—	—	0.23 (0.10-0.80)^f^	—	0.37 (0.13-1.06)
	15-19 (high)	—	—	0.22 (0.07-0.60)^e^	—	0.25 (0.08-0.81)^f^
	≥20 (very high)	—	—	0.25 (0.08-0.72)^e^	—	0.26 (0.08-0.82)^f^
**LSN size**						
	0	—	—	—	1.00	1.00
	1-2 (medium)	—	—	—	0.77 (0.39-1.37)	0.85 (0.47-1.54)
	≥3 (high)	—	—	—	0.32 (0.23-0.45)^e^	0.36 (0.27-0.50)^e^

^a^USC: unassisted smoking cessation.

^b^OR: odds ratio.

^c^OSN: ordinary social network.

^d^LSN: lower social network.

^e^*P*<.01.

^f^*P*<.05.

^g^Not applicable.

**Figure 1:**
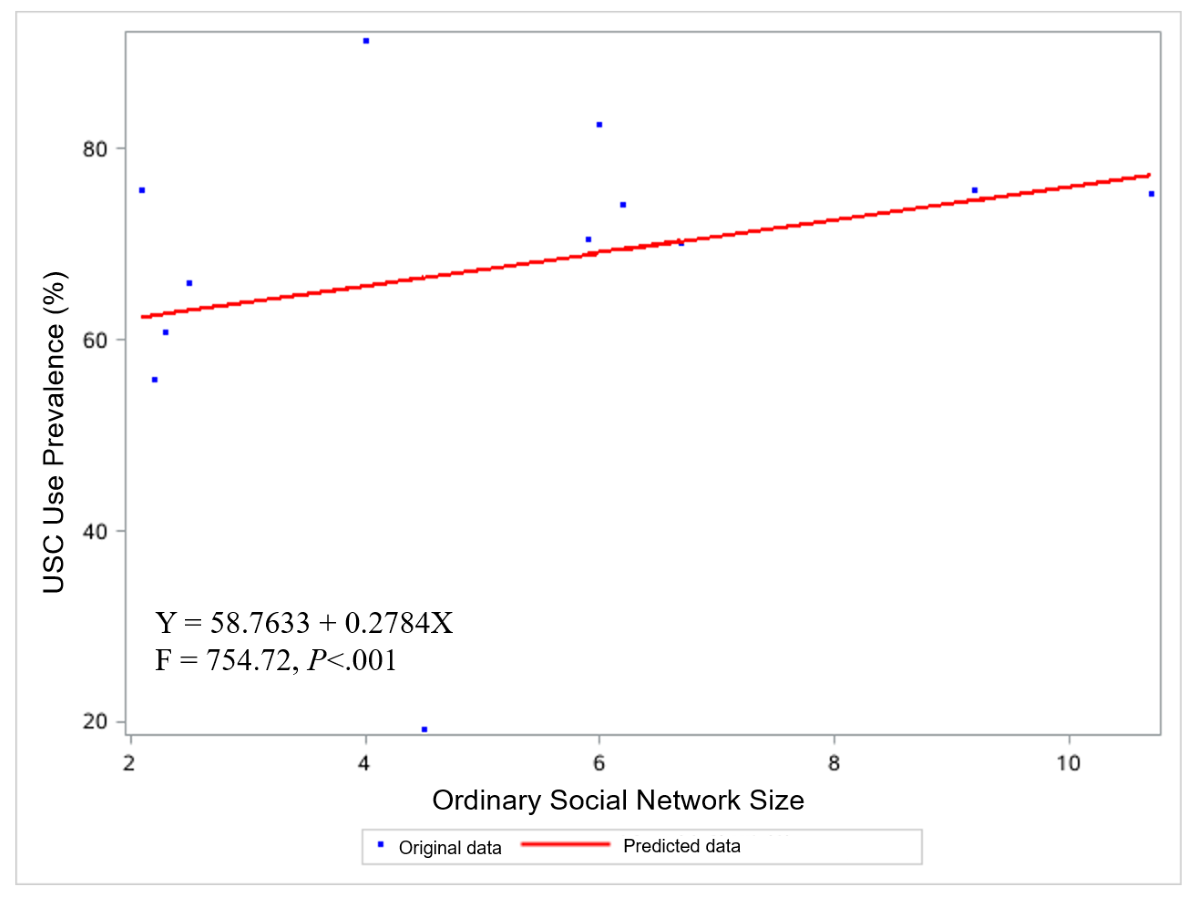
Scatter plot showing the relationship between city-level OSN size and the prevalence of USC adoption. OSN: ordinary social network; USC: unassisted smoking cessation.

**Figure 2:**
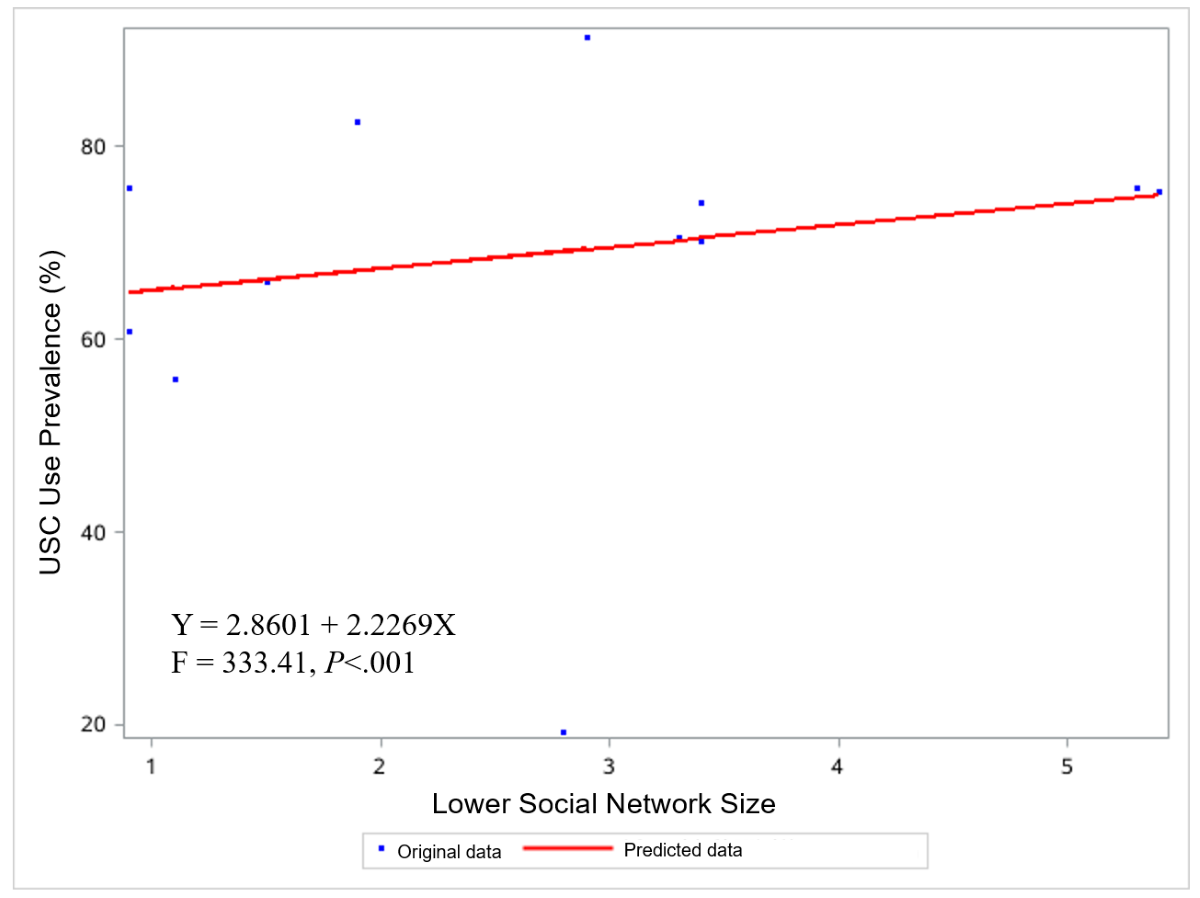
Scatter plot showing the relationship between city-level LSN size and the prevalence of USC adoption. OSN: lower social network; USC: unassisted smoking cessation.

The analysis revealed a significant positive dose-response relationship of city-level OSN and LSN sizes with the probability of USC adoption, as indicated by regression coefficients β=0.2784 (*P*<.01) and β=2.2269 (*P*<.01), respectively (see Figures 1 and 2).

**Figure 3:**
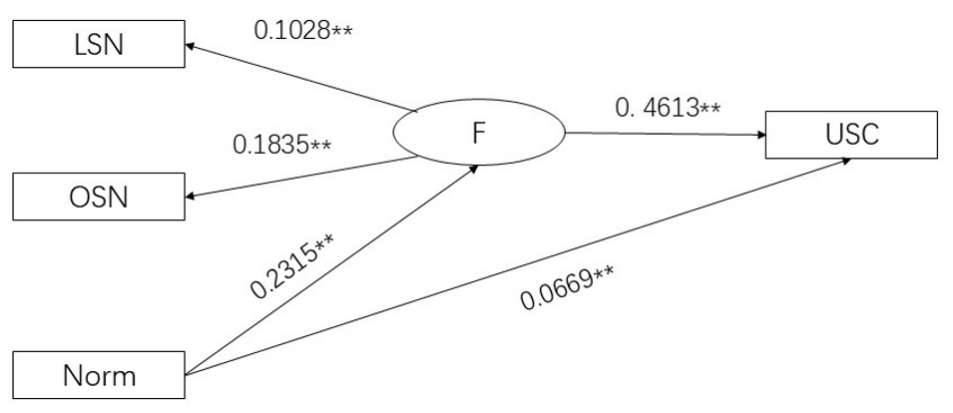
Results of SEM analysis. **P<.05. F: social network size; LSN: lower social network; Norm: subjective norm, perceived social pressure to engage in or refrain from a behavior; OSN: ordinary social network; SEM: structural equation modeling; USC: unassisted smoking cessation.

Table 3 presents the means (SDs) of the variables used in model construction. SEM analysis demonstrated good model fit, with GFI=0.976, NFI=0.954, and RMSEA=0.0063. Results also indicated that reference group norms had both direct effects on USC adoption (β=0.0669, *P*<.01) and indirect effects through OSNs and LSNs, with the indirect effect being β=0.2315×0.4613 (*P*<.01). In contrast, bigger OSN and LSN sizes also had a direct association with USC adoption (β=0.4613, *P*<.01), as illustrated in Figure 3.

**Table 3:** Means (SDs) of variables included in constructing models.

Variable	Mean (SD)
LSN^a^ size	4.05 (1.36)
OSN^b^ size	7.64 (2.41)
USC^c^	1.75 (1.69)
Norm^d^	1.55 (0.44)

^a^LSN: lower social network.

^b^OSN: ordinary social network.

^c^USC: unassisted smoking cessation.

^d^Norm: subjective norm, perceived social pressure to engage in or refrain from a behavior.

## Discussion

### Principal Findings

This study found that social networks are significantly related to USC adoption. China produces and consumes more tobacco than any other country in the world and as such experiences high levels of tobacco-related disease. Many studies have recently directly or indirectly referenced Chinese culture as a major contributor to China’s high tobacco usage [5]. In contrast to Western culture, Chinese culture has historically seen smoking as a form of social communication. The practices of gifting and sharing cigarettes are well accepted and pervasive across China [5]. The Chinese place a high value on interpersonal relationships and connections. These connections can be mutually beneficial. Central to the idea of “relationship” is the concept of reciprocity. If someone receives a gift but fails to reciprocate appropriately, that will not only lead to a loss of face but also threaten that person’s “relationship” and social capital. Accepting a cigarette can symbolize a person’s willingness to engage in a future business partnership, illicit or otherwise [38]. Given the importance of social networks, they need to be associated with smoking or quitting.

The findings of this study indicate that although HSNs have no significant association with USC adoption, both OSNs and LSNs are negatively related to the likelihood of adopting USC. Furthermore, this study found a significant positive dose-response relationship of city-level OSN and LSN sizes with the probability of adopting USC. These results suggest that the structure and composition of a smoker’s social network may play a crucial role in determining their smoking cessation strategies. According to TRA, reference group norms and social expectations significantly affect behavioral intentions. Social networks exert a strong effect on smoking behaviors and cessation outcomes, as individuals embedded in smoking-prevalent circles are more likely to maintain smoking habits or face challenges in quitting because of constant exposure to smoking cues, such as friends who smoke or social gatherings where tobacco use is normative. Those within OSN and LSN environments may experience stronger social reinforcement of smoking and fewer supportive cues for quitting, which, in turn, reduces their likelihood of successful USC. Peer influence in such networks often normalizes smoking and undermines self-initiated quitting efforts, especially when external interventions or alternative social incentives are lacking. Several mechanisms may further explain this pattern. First, the smoking cessation behaviors of socioeconomic groups are often more significantly affected by collective norms and the sharing of peer experiences. When an individual observes others around them successfully quitting smoking with the aid of cessation tools (eg, nicotine replacement products, quit lines, or the doctor’s advice), they are more likely to imitate this behavior. Thus, within OSNs and LSNs, the diffusion of smoking cessation assistance behaviors tends to be more powerful than quitting through willpower alone [22,39]. Moreover, individuals from socioeconomically disadvantaged groups tend to report lower self-efficacy in managing health behaviors, making them more inclined to seek professional help instead of relying solely on self-control [40,41]. Finally, these individuals also tend to experience a higher burden of health problems and therefore have more frequent interactions with medical and public health services [42]. Such increased contact may enhance their exposure to assisted smoking cessation programs, which helps explain why smokers embedded in OSNs and LSNs are more likely to seek professional support for smoking cessation and less likely to engage in USC. Taken together, these findings suggest that low-status social environments may influence smoking cessation behaviors, not simply through socioeconomic disadvantage, but also through network-based pathways that shape access to institutional and social resources.

This study found that reference group norms exert both a direct effect on USC and indirect effects through OSNs and LSNs. By integrating social network dynamics into TRA, this approach enriches its explanatory power and highlights the importance of interpersonal influences in shaping behavioral intentions [12,39]. Academically, the findings contribute to the literature on social influence and health behavior by demonstrating that normative pressures are not only transmitted directly but also mediated through network structures. Practically, the results provide valuable insights for intervention design: by identifying key network configurations and normative pathways, policymakers and health practitioners can more effectively promote smoking cessation behaviors. This underscores the role of social networks in embedding reference group norms within behavioral decision-making processes, suggesting that interventions targeting peer groups and social ties may yield greater success in reducing smoking prevalence.

In this study, 91.6% of those who quit smoking reported quitting without assistance, a percentage similar to that found in another study (93.1%) by the Chinese Center for Disease Control and Prevention (CDC) [26]. USC adoption is much higher in China than in the Western world. In Australia, Smith et al [43] found that between 54% and 69% of former smokers had quit without professional help, while 41%-58% of current smokers had tried to quit without assistance. In the United States, earlier research on successful attempts to quit smoking found that unassisted quit rates vary between 64% and 78% [44,45]. In Canada, a study found that Chinese immigrant smokers infrequently use cessation aids or services [46]. Furthermore, the prevalence of USC success was high (42.1%) in this study, which is also much higher than that in Western society [11,45]. This may reflect differences in cultural norms. Chinese culture, to a large extent, still adheres to agrarian social mores, which emphasize individual spirit and perseverance in coping with behavioral problems [12]. Thus, quitting smoking on their own rather than relying on professional assistance reflects an individual’s strength and willpower. Based on this cultural model for smoking cessation, decision makers need to adapt to the situation and actively promote USC among the Chinse population.

The findings reveal that socioeconomic and demographic factors play a complex role in the adoption of USC. Although initial analyses identified ethnicity, education level, and marital status as significant correlates of USC adoption, only education remained an independent predictor when these factors were examined concurrently in a multivariate model. Specifically, compared to individuals with an elementary school education or below, those with junior high school education showed a significantly lower adoption rate of USC. This suggests that the apparent associations of ethnicity and marital status with USC adoption may be largely confounded by or mediated through educational attainment. Higher education likely confers advantages in health awareness and behavioral capacity that enable individuals to attempt and succeed in quitting smoking without formal assistance, regardless of their ethnic background or marital status. This study found an increase in USC adoption with age, which aligns with findings from other studies as well [11,38]. This may reflect the fact that as individuals age, the prevalence of health problems may increase, leading to a greater need and motivation to quit smoking [5]. Older smokers grew up in an era of relative scarcity of material and medical resources, making them more inclined to rely on personal willpower rather than external aids to solve problems. Nicotine replacement therapy (NRT), quit lines, and digital health tools have only become widespread in recent decades. Older adults may be less familiar with these newer methods or skeptical about their safety and effectiveness, leading them to place greater trust in traditional, self-driven approaches to quitting smoking. It is interesting to note that in this study, the smoking amount was linked to the choice of unassisted quitting, and heavy smokers were less likely to quit smoking independently. This aligns with findings from other studies [11,45]. Perhaps this is because heavy smokers typically have higher nicotine dependence, which can hinder their ability to quit smoking independently, leading to a lower prevalence of USC adoption.

This study revealed that the smoking prevalence was 44.8%. Among smokers, 57.8% quit smoking, with 91.6% of them not using assistance. Among the USC users, 42.1% successfully achieved abstinence. This indicates that the USC success prevalence among smokers was 22.3%. According to the Chinese CDC survey of 2018, more than 308 million adults in China were current smokers [47]. Using the USC success prevalence from this study, approximately 68.7 million Chinese smokers could be projected to achieve abstinence if they try USC. A study [26] using direct USC numbers found that USC contributed to successful smoking cessation by approximately 70 million smokers in China. It is worth noting that the numbers from both sources are similar, indicating their reliability. It is indeed a massive number of those who quit smoking, and if this were to happen, it would produce positive changes in their health, lifestyle, and overall well-being. It is widely recognized that clinical and other individual-level smoking cessation methods are insufficient to fundamentally reduce smoking prevalence at the population level. Although such interventions can achieve high success rates among participants, their overall impact on national smoking rates remains limited, as they fail to reach the majority of smokers. More importantly, individual-oriented strategies often do not adequately address broader socioeconomic and cultural factors that contribute to sustained smoking behavior in China. These include aggressive marketing by the tobacco industry; social norms that tolerate, and even encourage, smoking by males; the use of cigarettes in social rituals; and the affordability of tobacco products. Without complementary population-level measures, such as those recommended by the WHO FCTC, including substantial tobacco tax increases, comprehensive bans on tobacco advertising, and the implementation of smoke-free laws, the effectiveness of stand-alone clinical interventions remains significantly constrained. Such policies are essential to reshaping social, economic, and environmental conditions to make smoking less attractive and accessible. This study provides evidence that USC adoption can effectively reduce the smoking prevalence in the population. However, this approach seems to have been overlooked by many Chinese health professionals and government officials. The WHO FCTC requires parties to promote tobacco cessation and implement effective measures to help tobacco users quit [4]. In China, smoking cessation assistance by health care professionals is common and receives considerable attention from the government. However, USC is often overlooked by tobacco control agencies, as they tend to consider it nonprofessional. USC can, in fact, be strengthened through professional strategies, such as implementing smoke-free environments, launching large-scale health education campaigns, and using media-based cessation interventions. Notably, engagement in USC may also heighten individuals’ awareness of, and interest in, professional smoking cessation support services. What we urgently need to do is improve the understanding of the effectiveness of USC and enhance professional services for USC to increase the number of people trying to quit smoking and to enhance USC success rates.

### Study Limitations

This study has a few limitations. First, as this study used a cross-sectional design, causal relationships cannot be established. Future longitudinal or experimental studies are needed to further explore causal mechanisms underlying these relationships. Although USC may promote attempts to quit smoking or short-term smoking cessation success, it does not necessarily lead to sustained long-term abstinence. The issue of long-term smoking cessation outcomes, along with comparisons between USC and other established smoking cessation support measures, particularly those aligned with Article 14 of the WHO FCTC, remains a critical area for future research.

Second, we assessed the social network size using the network scale-up method, which estimates the number of individuals a respondent knows. This approach has been widely applied in population studies and social network research to approximate the personal network size [28,29]. We acknowledge that this approach primarily captures the breadth of social connections rather than their depth, intensity, or emotional closeness. Although this method may not fully reflect the strength or quality of relationships, it provides a standardized and efficient means of estimating the network size across large populations. Given the scope and objectives of our study, this method was appropriate for our analytical framework. Nonetheless, we recognize the limitations of this approach and suggest that future research incorporate complementary measures, such as tie strength, frequency of interaction, or perceived support, to provide a more nuanced understanding of social networks.

Third, the occupational categories used in the LSN and HSN measures are relatively limited, as they were specifically designed to capture culturally recognized social groups within the Chinese context. Although this targeted approach facilitates the identification of low- and high-status networks, it may not fully encompass the broader diversity of participants’ social connections. Although reliability and validity analyses indicate strong psychometric performance, it remains possible that certain subpopulations were insufficiently represented. To enhance representativeness, future research should consider broadening the occupational scope of these measures.

Fourth, the OSN measure is based on the network scale-up method, which estimates the social network size by asking respondents how many people they know with certain common surnames. This approach has been widely used in social network research and has demonstrated validity in estimating general network exposure [48]. In our adaptation, we selected the most prevalent Chinese surnames to ensure cultural relevance. This method provides a practical proxy for the network size, but we recognize that relying solely on surname recognition may underestimate the complexity and richness of actual social networks, especially in urban or highly mobile populations.

### Conclusion

This study demonstrates that USC is the predominant method used by Chinese smokers. It further reveals that individuals embedded in OSNs or LSNs are less likely to adopt USC and that these networks exert indirect effects within the structure linking reference group norms and USC adoption, underscoring the influence of social environments on smoking cessation behavior. Mass media campaigns, public health initiatives, and the promotion of smoke-free environments are all effective ways to encourage the use of smoking cessation strategies, which lead to changes within social networks, ultimately resulting in shifts in network norms and USC adoption. For further tobacco control, policies should move beyond treatment provision toward reshaping social environments and network norms, as sustained reductions in smoking prevalence depend on cultural denormalization rather than clinical service expansion alone.

## Abbreviations

AIC: Akaike Information Criterion
CDC: Center for Disease Control and Prevention
EFA: exploratory factor analysis
FCTC: Framework Convention on Tobacco Control
GFI: goodness-of-fit index
HSN: higher social network
LSN: lower social network
NFI: normed fit index
OR: odds ratio
OSN: ordinary social network
RMSEA: root mean square error of approximation
SC: Schwarz Criterion
SEM: structural equation modeling
TRA: theory of reasoned action
TSN: total social network
USC: unassisted smoking cessation
VIF: variance inflation factor
WHO: World Health Organization

## Data Availability

The datasets used and analyzed during the study are available from the corresponding author upon reasonable request.

## References

[1] Schoberberger R, Böhm G, Schroeder Y (2015). Heavy dependent nicotine smokers--newfound lifestyle appreciation after quitting successfully. Experiences from inpatient smoking cessation therapy. Public Health.

[2] Tonstad S, Tønnesen P, Hajek P, Williams KE, Billing CB, Reeves KR, Varenicline Phase 3 Study Group (2006). Effect of maintenance therapy with varenicline on smoking cessation: a randomized controlled trial. JAMA.

[3] Hand S, Edwards S, Campbell IA, Cannings R (2002). Controlled trial of three weeks nicotine replacement treatment in hospital patients also given advice and support. Thorax.

[4] World Health Organization (2005). WHO Framework Convention on Tobacco Control.

[5] Yang T, Yang G (2012). Smoking cessation strategies in tobacco control in China. J Tuberc Lung Health.

[6] Jiang S, Yang T, Bullen C, Chen J, Yu L, Peng S, Rockett IRH (2021). Real-world unassisted quit success and related contextual factors: a population-based study of Chinese male smokers. Tob Control.

[7] Yan Y, Lin B, Xu Q, Xie H, Zeng X, Di X, Meng Z, Xiao L, Liu S (2023). Utilization of smoking cessation support among adults - 18 PLADs, China, 2020. China CDC Wkly.

[8] Smith AL, Chapman S, Dunlop SM (2015). What do we know about unassisted smoking cessation in Australia? A systematic review, 2005-2012. Tob Control.

[9] Soulakova JN, Crockett LJ (2017). Unassisted quitting and smoking cessation methods used in the United States: analyses of 2010-2011 tobacco use supplement to the current population survey data. Nicotine Tob Res.

[10] Yang T, Zhu Z, Barnett R, Zhang W, Jiang S (2019). Tobacco advertising, anti-tobacco information exposure, environmental smoking restrictions, and unassisted smoking cessation among Chinese male smokers: a population-based study. Am J Mens Health.

[11] Zhu S, Melcer T, Sun J, Rosbrook B, Pierce JP (2000). Smoking cessation with and without assistance: a population-based analysis. Am J Prev Med.

[12] Yang T (2025). Health Research: Social and Behavioral Theories and Methods, 3rd ed.

[13] Baer M (2010). The strength-of-weak-ties perspective on creativity: a comprehensive examination and extension. J Appl Psychol.

[14] Yang XY, Kelly BC, Yang T (2014). The influence of self-exempting beliefs and social networks on daily smoking: a mediation relationship explored. Psychol Addict Behav.

[15] Abel G, Plumridge L, Graham P (2009). Peers, networks or relationships: strategies for understanding social dynamics as determinants of smoking behaviour. Drugs: Educ Prev Policy.

[16] Fang X, Li X, Stanton B, Dong Q (2003). Social network positions and smoking experimentation among Chinese adolescents. Am J Health Behav.

[17] Pearson M, Michell L (2000). Smoke rings: social network analysis of friendship groups, smoking and drug-taking. Drugs: Educ Prev Policy.

[18] Seo D, Huang Y (2012). Systematic review of social network analysis in adolescent cigarette smoking behavior. J Sch Health.

[19] Ross B, Tingzhong Y, Xiaozhao Y (2021). Smoking Environments in China: Challenges for Tobacco Control.

[20] Pan Z (2004). Socioeconomic predictors of smoking and smoking frequency in urban China: evidence of smoking as a social function. Health Promot Int.

[21] Yang T, Fisher KJ, Li F, Danaher BG (2006). Attitudes to smoking cessation and triggers to relapse among Chinese male smokers. BMC Public Health.

[22] Christakis NA, Fowler JH (2008). The collective dynamics of smoking in a large social network. N Engl J Med.

[23] Hoffman BR, Sussman S, Unger JB, Valente TW (2006). Peer influences on adolescent cigarette smoking: a theoretical review of the literature. Subst Use Misuse.

[24] Montes F, Blanco M, Useche AF, Sanchez-Franco S, Caro C, Tong L, Li J, Zhou H, Murray JM, Sarmiento OL, Kee F, Hunter RF (2023). Exploring the mechanistic pathways of how social network influences social norms in adolescent smoking prevention interventions. Sci Rep.

[25] Sohn M, Moon D, Kim J (2023). A longitudinal cohort study of adolescent social network positions and lifetime daily smoking and nicotine dependence. Youth Soc.

[26] (2011). World Health Organization. Global adult tobacco survey (GATS) China 2010 country report.

[27] Williams MB, Beebe LA, Neas BR (2015). State-level correlates of unassisted quit attempts and success. J Okla State Med Assoc.

[28] Maltiel R, Raftery AE, McCormick TH, Baraff AJ (2015). Estimating population size using the network scale up method. Ann Appl Stat.

[29] McCormick TH, Salganik MJ, Zheng T (2010). How many people do you know?: efficiently estimating personal network size. J Am Stat Assoc.

[30] Fabrigar LR, Wegener DT, MacCallum RC, Strahan EJ (1999). Evaluating the use of exploratory factor analysis in psychological research. Psychol Methods.

[31] Hair J, Lovric M (2011). Multivariate data analysis: an overview. International Encyclopedia of Statistical Science.

[32] Stevens J (1992). Applied Multivariate Statistics for the Social Sciences, 2nd ed.

[33] Taber KS (2017). The use of Cronbach’s alpha when developing and reporting research instruments in science education. Res Sci Educ.

[34] Gareth J, Daniela W, Trevor H, Tibshirani R (2021). An Introduction to Statistical Learning.

[35] Allison P (2012). Logistic Regression Using SAS: Theory and Application, 2nd ed.

[36] Sommet N, Morselli D (2017). Keep calm and learn multilevel logistic modeling: a simplified three-step procedure using Stata, R, Mplus, and SPSS. Int Rev Soc Psychol.

[37] Hu L, Bentler PM (1999). Cutoff criteria for fit indexes in covariance structure analysis: conventional criteria versus new alternatives. Struct Equ Model.

[38] Wank D, Davis D (2000). Cigarettes and domination in Chinese business networks: institutional change during the market transition. The Consumer Revolution in Urban China.

[39] Valente TW (2010). Social Networks and Health: Models, Methods, and Applications.

[40] Bandura A (1977). Self-efficacy: toward a unifying theory of behavioral change. Psychol Rev.

[41] Hiscock R, Bauld L, Amos A, Fidler JA, Munafò M (2012). Socioeconomic status and smoking: a review. Ann N Y Acad Sci.

[42] Brown T, Platt S, Amos A (2014). Equity impact of European individual-level smoking cessation interventions to reduce smoking in adults: a systematic review. Eur J Public Health.

[43] Smith AL, Carter SM, Chapman S, Dunlop SM, Freeman B (2015). Why do smokers try to quit without medication or counselling? A qualitative study with ex-smokers. BMJ Open.

[44] Cokkinides VE, Ward E, Jemal A, Thun MJ (2005). Under-use of smoking-cessation treatments: results from the National Health Interview Survey, 2000. Am J Prev Med.

[45] Shiffman S, Brockwell SE, Pillitteri JL, Gitchell JG (2008). Use of smoking-cessation treatments in the United States. Am J Prev Med.

[46] Mao A, Bottorff JL (2017). A qualitative study on unassisted smoking cessation among Chinese Canadian immigrants. Am J Mens Health.

[47] Xiao L, Nan Y, Di XB, Meng ZD (2022). Study on smoking behavior and its changes among Chinese people aged 15 years and above in 2018. Zhonghua Liu Xing Bing Xue Za Zhi.

[48] Clay-Warner Jody, Kawashima Tenshi, Edgemon Timothy G (2022). Measure of personal network size using the known population method: a methodological guide. Am J Public Health.

